# Oncolytic viruses are a recent breakthrough in oncoimmune treatment: recent insights – correspondence

**DOI:** 10.1097/MS9.0000000000000278

**Published:** 2023-03-14

**Authors:** Ruhul Amin, Bapi R. Sarkar, Prosanta Pal, Amitava Roy, Talha B. Emran

**Affiliations:** a Faculty of Pharmaceutical Science, Assam Down Town University, Panikhaiti, Guwahati, Assam, India; b Department of Pharmaceutical technology, University of North Bengal, Darjeeling, West Bengal, India; c Department of Pharmacy, BGC Trust University Bangladesh, Chittagong, Bangladesh; d Department of Pharmacy, Faculty of Allied Health Sciences, Daffodil International University, Dhaka, Bangladesh

Oncolytic viruses have many anticancer strategies. Genetically modified viruses may more effectively infect cancer cells than normal cells^1^. The CF33 vaccinia (pox) chimeric virus was developed by Professor Yuman Fong^2^. Vaccinia has fast cell-to-cell replication and dissemination that is well understood, yet it does not integrate into the host genome. It is effective against a wide variety of tumor cell lines and has a potent cytolytic effect. It may be used as an oncolytic drug and a vehicle for delivering genes for gene therapy. Genomic sequences from different vaccinia virus strains were combined to create CF33, a novel virus that is both safer and more effective than previous versions. With the use of in-vivo imaging and targeted irradiation, Vaxinia, a CF33 modified to express the human sodium-iodide symporter (hNIS) gene, has been developed. CF33, ‘armed’ with hNIS and anti-PD-L1 genes, allowing for improved anticancer immunotherapy, is known as CHECKVacc^3^.

Numerous preclinical studies have shown the treatment’s safety, and evidence of both a local and systemic antitumor effect has emerged. Using CF33, it would be able to enhance the clinical advantages and quality of life for patients with malignancies that are now untreatable with standard therapy methods. Phase 1 clinical studies will use CF33-hNIS, a new chimeric orthopox virus, either as monotherapy or in combination with pembrolizumab to evaluate the safety and effectiveness of the treatment regimens and the immunological alterations in the tumor microenvironment^4^.

Gene therapy Talimogene Laherparepvec (T-VEC) overexpresses granulocyte macrophage colony-stimulating factor by using an attenuated herpes simplex virus 1 virus, which is then used in dendritic cell-mediated antigen presentation studies. Patients with advanced, nonresectable melanoma who received intratumoral granulocyte macrophage colony-stimulating factor alone had a lower rate of long-term response compared to those who received T-VEC^5^. The US Food and Drug Administration has authorized the use of this drug for the treatment of patients with unresectable or advanced melanoma who have metastasized to localized areas of skin or lymph nodes and have only little visceral disease.

Oncolytic virus injection may enhance the effects of checkpoint inhibitors by enhancing CD8^+^ T-cell infiltration and interferon-gamma signaling and by upregulating PD-L1 in the microenvironment^6^. The response rate to ipilimumab with T-VEC was significantly greater (39%) than the response rate to ipilimumab alone (18%) in a randomized study^7^. Responses to T-VEC plus pembrolizumab in melanoma patients were shown regardless of pre-existing immune infiltrate, according to results from a phase 1 study^6^.

In addition to the encouraging findings from the preclinical studies, we are very excited to explore the possibilities presented by the oncolytic virotherapy platform comprised of VAXINIA and T-VEC.

## Ethical approval

This case report has been reported in line with the Case Report (CARE) guidelines.

## Consent

Not applicable.

## Sources of funding

None.

## Author contribution

R.A.: conceptualization, data curation, writing – original draft preparation, writing – reviewing and editing. B.R.S., P.P., and A.R.: data curation, writing – original draft preparation, writing – reviewing and editing. T.B.E.: writing – reviewing and editing, visualization, supervision. All the authors confirm the concept, design, data collection and analysis and interpretation, and writing are our own. The authors confirm there are no other contributors.

## Conflicts of interest disclosure

The authors declare that they have no financial conflict of interest with regard to the content of this report.

## Research registration unique identifying number (UIN)

None.

## Guarantor

Talha Bin Emran, PhD, Associate Professor, Department of Pharmacy, BGC Trust University Bangladesh, Chittagong 4381, Bangladesh. Tel: +880 303 356 193, Fax: +880 312 550 224. https://orcid.org/0000-0003-3188-2272. E-mail: talhabmb@bgctub.ac.bd

## Provenance and peer review

Not commissioned, externally peer reviewed.

## References

[1] de Gruijl TD Janssen AB van Beusechem VW . Arming oncolytic viruses to leverage antitumor immunity. Expert Opin Biol Ther 2015;15:959–971. 25959450 10.1517/14712598.2015.1044433

[2] Woo Y Zhang Z Yang A . Novel chimeric immuno-oncolytic virus CF33-hNIS-antiPDL1 for the treatment of pancreatic cancer. J Am Coll Surg 2020;230:709–717. 32032721 10.1016/j.jamcollsurg.2019.12.027 PMC8787938

[3] Yuan Y Zhang J Kessler J . Phase I study of intratumoral administration of CF33-HNIS-antiPDL1 in patients with metastatic triple negative breast cancer; 2022.

[4] Henderson E . First patient dosed in Phase 1 clinical trial evaluating the safety of CF33-hNIS against solid tumors. May 18, 2022.

[5] Ferrucci PF Pala L Conforti F . Talimogene laherparepvec (T-VEC): an intralesional cancer immunotherapy for advanced melanoma. Cancers (Basel) 2021;13. 10.3390/cancers13061383 PMC8003308 33803762

[6] Ribas A Dummer R Puzanov I . Oncolytic virotherapy promotes intratumoral T cell infiltration and improves anti-PD-1 immunotherapy. Cell 2017;170:1109–1119.e1110. 28886381 10.1016/j.cell.2017.08.027 PMC8034392

[7] Chesney J Puzanov I Collichio F . Randomized, open-label phase II study evaluating the efficacy and safety of talimogene laherparepvec in combination with ipilimumab versus ipilimumab alone in patients with advanced, unresectable melanoma. J Clin Oncol 2018;36:1658–1667. 28981385 10.1200/JCO.2017.73.7379 PMC6075852

